# Cytomegalovirus infection is a risk factor for tuberculosis disease in infants

**DOI:** 10.1172/jci.insight.130090

**Published:** 2019-12-05

**Authors:** Julius Müller, Rachel Tanner, Magali Matsumiya, Margaret A. Snowden, Bernard Landry, Iman Satti, Stephanie A. Harris, Matthew K. O’Shea, Lisa Stockdale, Leanne Marsay, Agnieszka Chomka, Rachel Harrington-Kandt, Zita-Rose Manjaly Thomas, Vivek Naranbhai, Elena Stylianou, Stanley Kimbung Mbandi, Mark Hatherill, Gregory Hussey, Hassan Mahomed, Michele Tameris, J. Bruce McClain, Thomas G. Evans, Willem A. Hanekom, Thomas J. Scriba, Helen McShane, Helen A. Fletcher

**Affiliations:** 1 The Jenner Institute, Nuffield Department of Medicine, University of Oxford, Oxford, United Kingdom.; 2 Aeras, Rockville, Maryland, USA.; 3 London School of Hygiene & Tropical Medicine, London, United Kingdom.; 4 The Kennedy Institute and; 5 Wellcome Trust Centre for Human Genetics, University of Oxford, Oxford, United Kingdom.; 6 South African Tuberculosis Vaccine Initiative, Institute of Infectious Disease and Molecular Medicine & Division of Immunology, Department of Pathology, University of Cape Town, South Africa.

**Keywords:** Inflammation, Vaccines, NK cells, Tuberculosis

## Abstract

Immune activation is associated with increased risk of tuberculosis (TB) disease in infants. We performed a case-control analysis to identify drivers of immune activation and disease risk. Among 49 infants who developed TB disease over the first 2 years of life, and 129 healthy matched controls, we found the cytomegalovirus-stimulated (CMV-stimulated) IFN-γ response to be associated with CD8^+^ T cell activation (Spearman’s rho, *P* = 6 × 10^–8^). A CMV-specific IFN-γ response was also associated with increased risk of developing TB disease (conditional logistic regression; *P* = 0.043; OR, 2.2; 95% CI, 1.02–4.83) and shorter time to TB diagnosis (Log Rank Mantel-Cox, *P* = 0.037). CMV^+^ infants who developed TB disease had lower expression of NK cell–associated gene signatures and a lower frequency of CD3^–^CD4^–^CD8^–^ lymphocytes. We identified transcriptional signatures predictive of TB disease risk among CMV ELISpot–positive (area under the receiver operating characteristic [AUROC], 0.98, accuracy, 92.57%) and –negative (AUROC, 0.9; accuracy, 79.3%) infants; the CMV^–^ signature was validated in an independent infant study (AUROC, 0.71; accuracy, 63.9%). A 16-gene signature that previously identified adolescents at risk of developing TB disease did not accurately classify case and control infants in this study. Understanding the microbial drivers of T cell activation, such as CMV, could guide new strategies for prevention of TB disease in infants.

## Abstract 2

A case control analysis identifies an association of cytomegalovirus (CMV) with increased risk of developing tuberculosis in infants.

## Graphical abstract

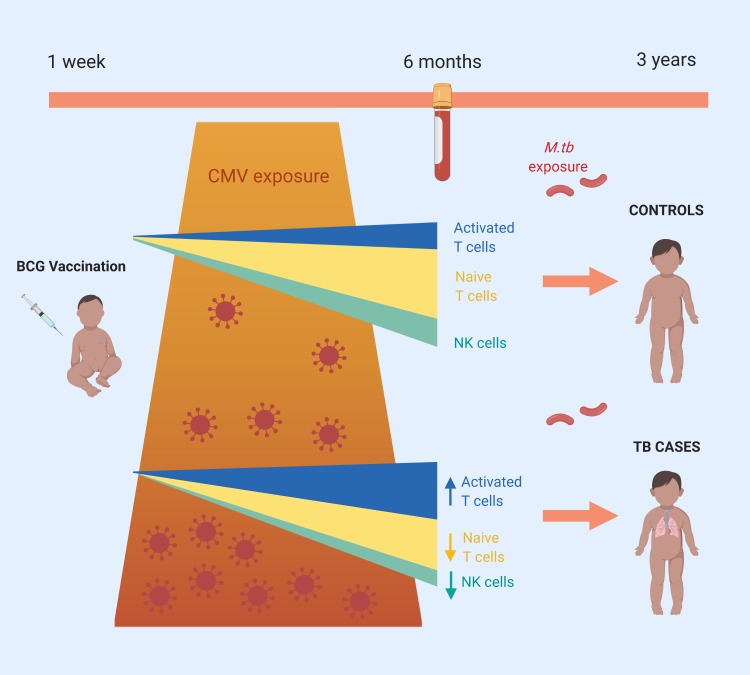

## Introduction

There are an estimated 1 million cases of childhood tuberculosis (TB) each year and in 2015, 210,000 children died of TB (1). Children with TB are difficult to diagnose and treat and are at risk of severe disease (2). The need for improved strategies to control childhood TB has led to studies to identify risk factors for TB disease in children. The longitudinal data collected during infant TB vaccine efficacy trials in South Africa with the vaccines Bacille Calmette-Guérin (BCG) and Modified Vaccinia Virus Ankara expressing Antigen 85A (MVA85A) (3, 4) have enabled the identification of correlates of TB disease risk in infants (5, 6). Using samples collected from 4- to 6-month-old infants at enrollment into the MVA85A efficacy trial in the Western Cape Province of South Africa (4), we reported that CD4^+^ T cell activation (measured as HLA-DR expression) was associated with increased risk of TB disease over the following 3 years of life (6). This finding was validated in an independent cohort of *Mycobacterium tuberculosis*–infected (*M.tb*–infected) adolescents (6). The consequences of chronic T cell activation have not been previously explored in the context of TB but are well described in HIV and include risk of acquisition of infection and disease progression in children (7–10) and adults (11–15). Recognition that T cell activation is a feature of HIV immunopathogenesis is guiding the development of new interventions for the management of HIV, such as the use of statins or Maraviroc as an adjunct to antiretroviral therapy (ART) or switching ART regimens (16–18).

Sustained T cell activation and dysfunction of antigen specific T cells can result from chronic exposure to antigen from persistent viral or bacterial infections (19). Human cytomegalovirus (CMV) and Epstein-Barr virus (EBV) are known drivers of T cell activation (12, 15), and CMV prevalence is high in developing countries — in particular, sub-Saharan Africa, where 85% of infants are infected by 12 months of age (reviewed in ref. 20). Recent epidemiological evidence supports a role for CMV in the etiology of TB with notable similarity in the age-sex distribution between the 2 infections (21). Rates of CMV infection were higher among TB patients than healthy volunteers in studies of both Nigerian adults and Russian children and adolescents (22, 23). In a rural Ugandan cohort, higher CMV exposure (measured by CMV IgG levels) was associated with active TB disease and lower levels of anti-mycobacterial antibodies (24, 25).

We previously reported that CD4^+^ T cell activation in infants and adolescents was associated with increased risk of developing TB disease (6). Here, we aimed to understand the drivers of this immune activation in the infant population — specifically whether viral infection with CMV or EBV may play a role. We used transcriptional analysis to further explore the pathways associated with immune activation and viral infection. Improved understanding of the causes and impact of T cell activation could transform future approaches to protect against TB, including the use of vaccines, antibiotics, or antivirals, as well as protective behaviors to reduce the burden of chronic microbial drivers.

## Results

**Figure 1:**
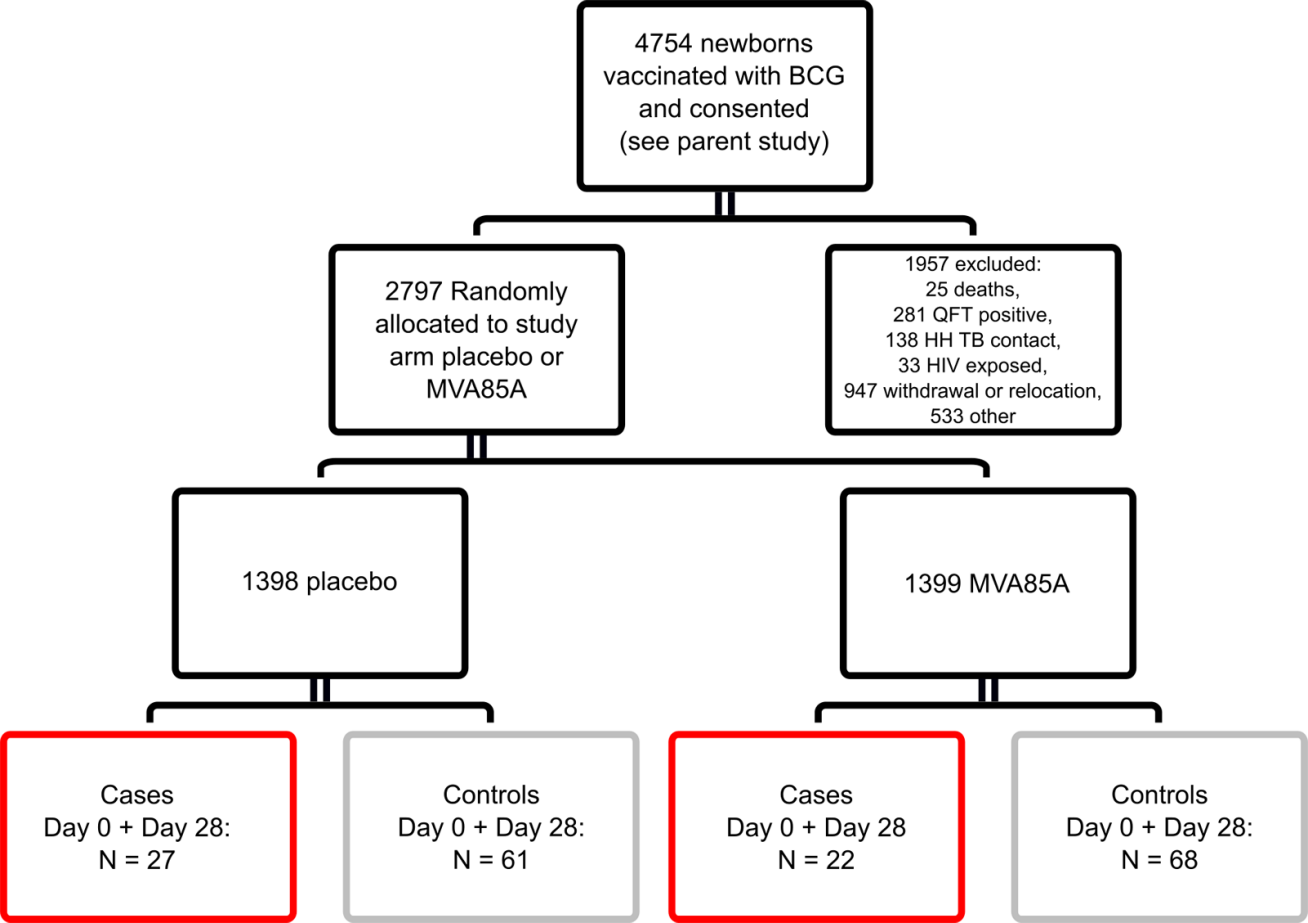
Study design for immune correlates analysis. Infants who were enrolled in an efficacy trial of the candidate TB vaccine MVA85A were included in this study (4). Infants were randomized at 16–24 weeks of age to receive a single intradermal dose of MVA85A or placebo (Candin, a candida skin test antigen) (4). Boxes indicate the number of case infant (red) or control infant (gray) samples available for combined Day 0 (D0) and D28 analysis. Analysis was restricted to infants where a frozen PBMC sample was available and live cells in PBMC were > 50% (or PHA IFN-γ ELISPOT ≥ 1000 SFC/million), as well as to infants where a sample was available for analysis from both the D0 and D28 time points. Control infants were excluded if the corresponding matched case was not in the analysis. QFT, QuantiFERON-TB Gold test; HH, household.

*Activated CD8^+^ T cells are associated with TB disease risk and correlate with CMV-specific IFN-γresponse*. Infants who were enrolled in an efficacy trial of the candidate TB vaccine MVA85A were included in this study (4) (Figure 1). All infants received BCG within 7 days of birth. Infants were screened at 4–6 months of age, and only those with clinical parameters within the normal range who were HIV- and *M.tb*–uninfected and without known TB exposure were included in the trial. CMV and EBV analysis were conducted on a blood sample taken at this time point. Infants were randomized to receive a single intradermal dose of MVA85A or placebo (Candin, a candida skin test antigen) (4). In contrast to our previous analysis, which focused on the sample collected at enrollment (day 0; D0), samples collected from 2 time points, D0 and D28 following MVA85A or placebo were combined for testing of both cellular parameters and transcriptional signatures associated with risk of TB disease. We have shown that the correlates of risk measured in our previous study were not affected by intervention (MVA85A or placebo) (6). Only infants for whom a sample was available at both D0 and D28 were included in the analysis (Figure 1).

Previously, we identified an association between TB disease risk over the first 2 years of life and the frequency of activated HLA-DR^+^CD4^+^ T cells at age 4–6 months (6). We also found that the magnitude of BCG-specific IFN-γ–expressing cells and levels of anti–Ag85A IgG were associated with reduced risk of TB disease (6). In our present analysis, with combined D0 and D28 samples from 49 cases and 129 controls (Figure 1), we confirmed our previous finding of TB risk associating with HLA-DR^+^CD4^+^, and reduced TB risk associating with BCG-specific IFN-γ, and anti–Ag85A IgG (Table 1). In addition, we found frequencies of HLA-DR^+^CD8^+^ T cells to be associated with TB risk (conditional logistic regression [CLR]; OR, 1.34; 95% CI, 1.08–1.67; *P* = 0.008; FDR, 0.092) (Table 1). The magnitude of PPD-stimulated IFN-γ–expressing cells measured by ELISpot were also associated with reduced TB risk (OR, 0.71; 95% CI, 0.51–0.98; *P* = 0.037; FDR, 0.2) (Table 1).

**Figure 2:**
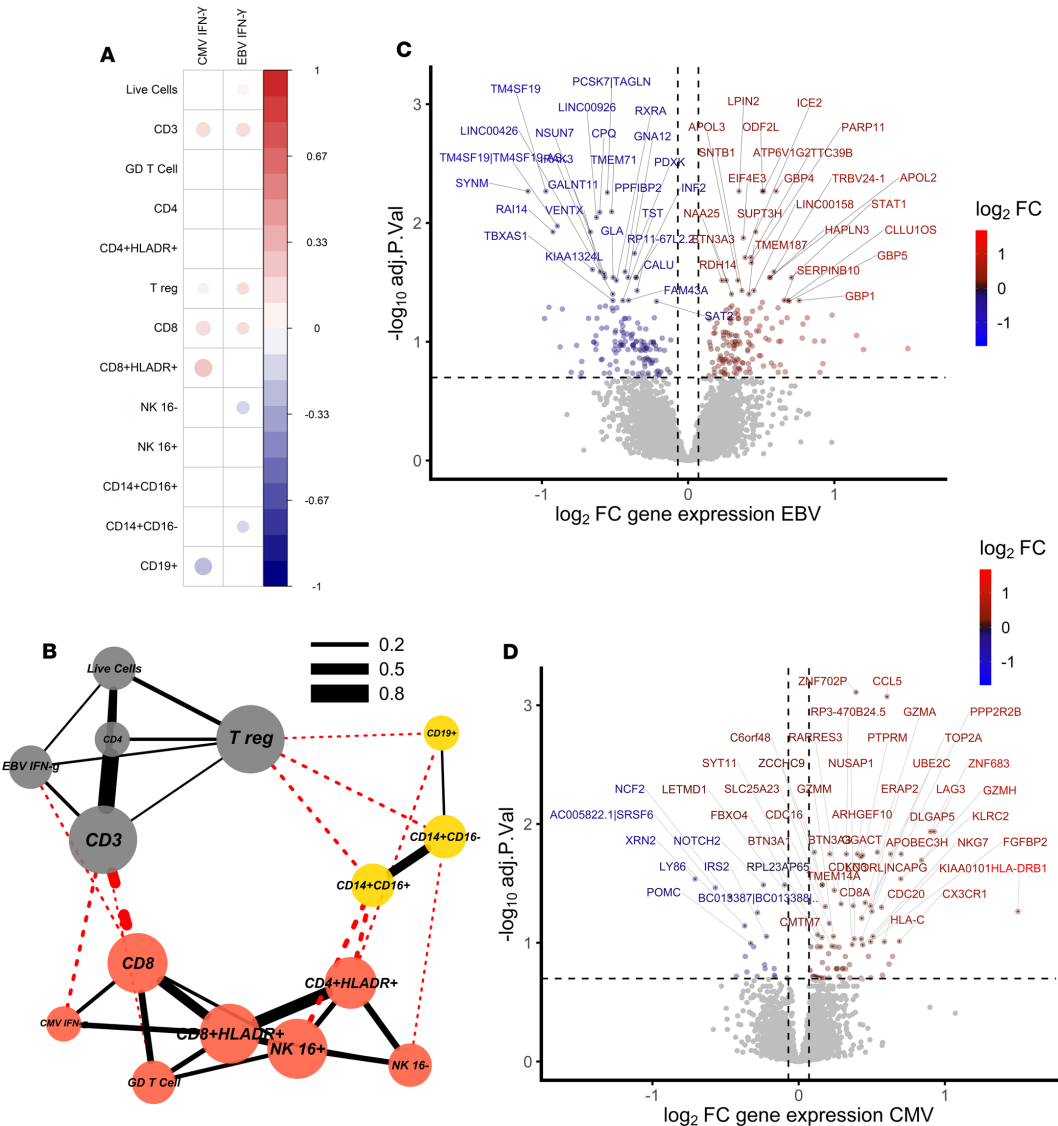
CMV infection is associated with CD8^+^ T cell activation in South African infants. (**A**) Correlation matrix of significantly (Spearman’s rho, *P* < 0.05) correlated cell populations and IFN-γ ELISpot responses to EBV and CMV using cellular data. The magnitude of the CMV-specific IFN-γ ELISpot response correlated with the frequency of activated CD8^+^ T cells. (**B**) Network of positively correlating cell populations using cellular data (Spearman’s rho, *P* < 0.05) showing 3 clusters dominated by activated T cells with CMV, CD3^+^ T cells with EBV, and monocytes with B cells (node color indicates cluster membership using clusters defined by ref. 26; red, clustering with CMV response; gray, clustering with EBV response; and yellow, clustering with myeloid cells). Red lines indicate between-cluster correlations, and black lines within-cluster correlations. Line width indicates the correlation coefficient. (**C**) Volcano plot using transcriptomic data showing magnitude and significance of differential expression between EBV^+^ and EBV^–^ infants, where blue indicates genes that are downregulated in EBV^+^ infants and red indicates genes that are upregulated in EBV^+^ infants. (**D**) CMV–strongly positive (ELISpot > 100 SFC/million) and CMV^–^ infants, where blue indicates genes that are downregulated in CMV^+^ infants and red indicates genes that are upregulated in CMV^+^ infants. Gray indicates genes for which expression is unchanged. The top 50 significant genes are labeled, and horizontal and vertical dashed lines indicate 20% FDR and 5% change in gene expression, respectively.

We analyzed CMV-specific and EBV-specific IFN-γ ELISpot responses for evidence of an association between viral infection and T cell activation in our infant cohort. Frequencies of activated CD8^+^ T cells correlated with the magnitude of the CMV-specific IFN-γ ELISpot response (Spearman’s rho, *P* = 6 × 10^–8^, Figure 2A), suggesting that CMV infection (as defined by a positive CMV-specific T cell response) is associated with CD8^+^ T cell activation in this infant cohort. A network representation of positively correlating cell populations (26) (Spearman’s rho, *P* < 0.05) revealed 3 clusters dominated by activated CD8^+^ and activated CD4^+^ T cells with CMV-specific IFN-γ ELISpot response, CD3^+^ T cells with EBV-specific IFN-γ ELISpot response, and monocytes with B cells (Figure 2B).

EBV had a strong effect on the blood transcriptome, with 296 genes differentially expressed between infants with positive and negative EBV responses (Figure 2C). CCL8, CXCL10, and IFIT3 had the greatest fold increase in expression in EBV^+^ infants, indicating strong induction of a Type I/II IFN response, although we did not see a correlation between EBV ELISpot response and T cell activation in this study (Supplemental Table 1; supplemental material available online with this article; https://doi.org/10.1172/jci.insight.130090DS1).

The impact of CMV on the blood transcriptome was smaller, with only 14 genes differentially expressed between CMV^+^ and CMV^–^ infants (ELISpot > 17 spot-forming cells [SFC]/million), although there were 103 differentially expressed transcripts between CMV–strongly positive (ELISpot > 100 SFC/million) and CMV^–^ infants (Figure 2D and Supplemental Tables 2, A and B). Differentially expressed transcripts included HLA-DRB1, ZNF683, LAG3, and KLRC2 (NKG2C). ZNF683 is an important paralog of PRDM1 and, together with CCL5, has been shown to be highly induced in human CMV-specific CD8^+^ T cells when compared with naive CD8^+^ T cells (27). LAG3 is expressed on activated CD8^+^ and CD4^+^ T cells, and LAG3^+^CD8^+^ T cells are found in CMV infection (27, 28). NKG2C^+^ NK cells are expanded in response to CMV infection, although CMV infection can also induce the expression of NKG2C on CD8^+^ T cells (29–31).

Expression levels of transcripts from total peripheral blood mononuclear cells (PBMC) of infants with a CMV ELISpot response (>100 SFC/million) were correlated with expression levels of transcripts published from CD8^+^ T cells isolated from adults at different stages of CMV infection (27). The highly significant correlation of infant transcripts with CMV-specific CD8^+^ T cell transcripts from adults confirms the prominence of an activated CD8^+^ T cell response in unstimulated PBMC of CMV ELISpot–positive infants (Supplemental Figure 1, A and B).

Our cellular data suggest that CMV IFN-γ responses are associated with activated HLA-DR^+^CD8^+^ T cells, and transcriptional analysis supports the presence of an activated CD8^+^ T cell phenotype among total, unstimulated PBMC from CMV IFN-γ ELISpot–positive infants.

### CMV antigen–specific T cell responses, measured up to 3 years prior to detection of TB, associate with risk of TB disease in infants.

**Figure 3:**
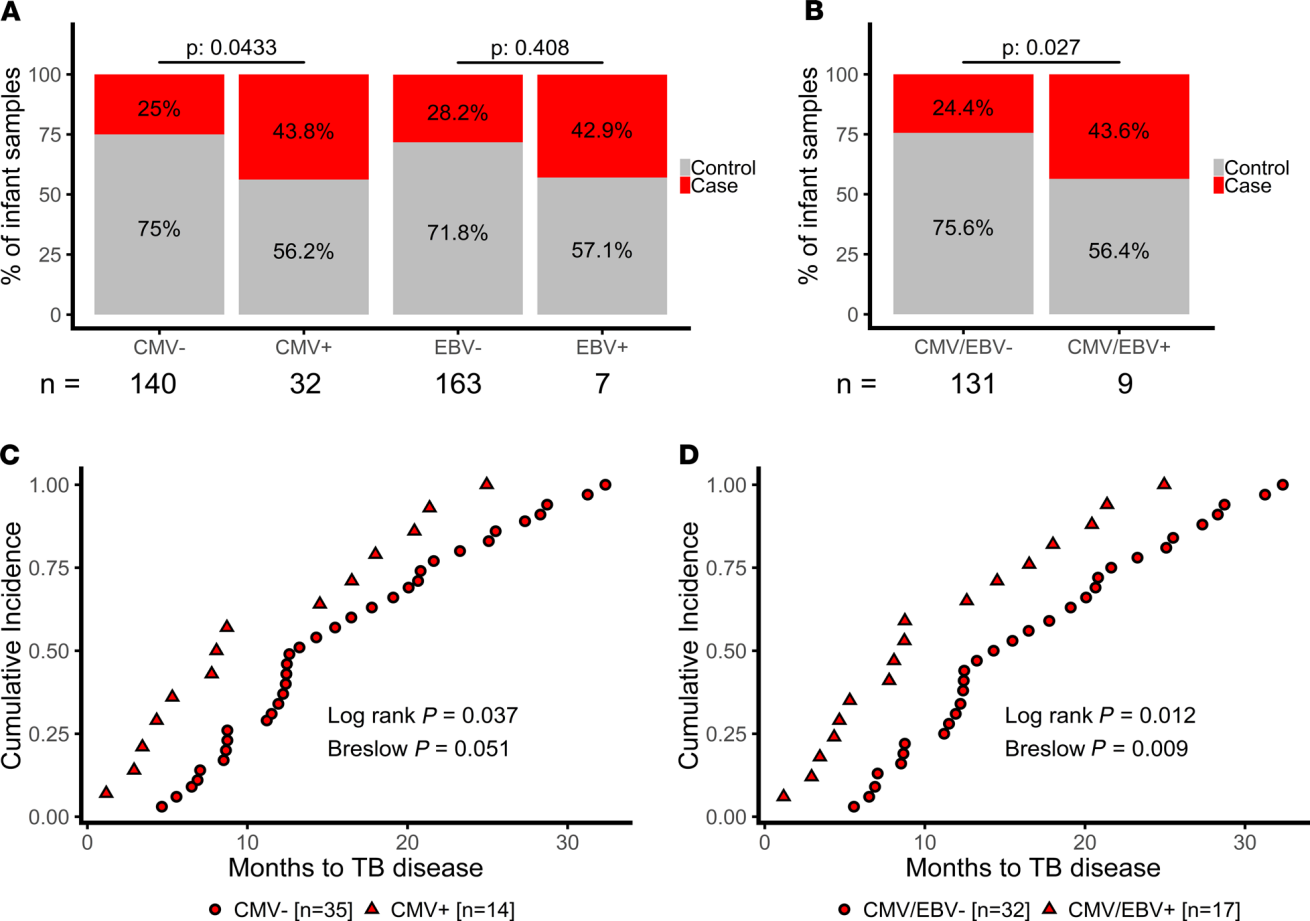
CMV^+^ infants are at increased risk of developing TB disease. (**A**) We saw a higher proportion of case (red) infants among CMV^+^ (*n* = 14 of 32) when compared with CMV^–^ infants (*n* = 35 of 140), and there was no significant enrichment for cases among EBV^+^ infants (*n* = 3 of 7 compared with *n* = 46 of 163), although infants positive for either CMV or EBV (**B**) were at increased risk (*n* = 17 of 39 compared with 32 of 131). (**C**) CMV^+^ TB case infants (red triangles, *n* = 14) developed TB disease earlier in follow-up when compared with CMV^–^ infants (red circles, *n* = 35), and (**D**) TB case infants positive for either CMV or EBV (red triangles, *n* = 17) developed TB disease earlier than CMV^–^/EBV^–^ infants (red circles, *n* = 32).

CMV is associated with increased risk of HIV infection and disease progression (12, 13, 15). To determine if viral infection is associated with TB risk in our infant cohort, we analyzed CMV-specific and EBV-specific IFN-γ ELISpot responses in cases and controls. Infants dual-positive for both EBV and CMV (*n* = 3) were excluded from the analysis. When CMV status was treated as a binary variable, CMV positivity (defined as a CMV-specific IFN-γ ELISpot response > 17 SFC/million at either time point), measured up to 3 years prior to TB detection, was associated with increased risk of TB disease (CLR; *P* = 0.043; OR, 2.22; 95% CI, 1.02–4.83) (Figure 3A). An ELISpot response to EBV alone was not associated with risk (Figure 3A), although a combined CMV and EBV response was associated with increased risk of TB disease (CLR; *P* = 0.025; OR, 2.3; 95% CI, 1.11–4.79) (Figure 3B). To further explore this association, we analyzed time to TB diagnosis in TB cases. Infants with a positive CMV or CMV^+^/EBV^+^ ELISpot response developed TB earlier than negative infants (Log Rank Mantel-Cox, *P* = 0.037; Figure 3, C and D).

We analyzed data by vaccine group and saw no evidence that infants with positive CMV or CMV^+^/EBV^+^ ELISpot responses who were immunized with MVA85A were at greater or lower risk of TB disease when compared with infants with negative CMV ELISpot responses who were immunized with MVA85A (*P* = 0.8355 for CMV and *P* = 0.9177 for CMV/EBV).

Interestingly, there was almost no overlap in differentially expressed genes between CMV^+^ and EBV^+^ infants with only 7 genes significantly differentially expressed in both comparisons at an FDR of 20% (Supplemental Figure 2).

A CMV-specific IFN-γ response measured at 4–6 months of age, up to 3 years before disease is detected, was a risk factor for the development of TB disease in South African infants, and this risk was greatest during the first 10 months of follow-up.

### Transcriptional evidence of activated T cells, Type I IFN responses, and NK cells in infants up to 3 years prior to detection of TB.

**Figure 4:**
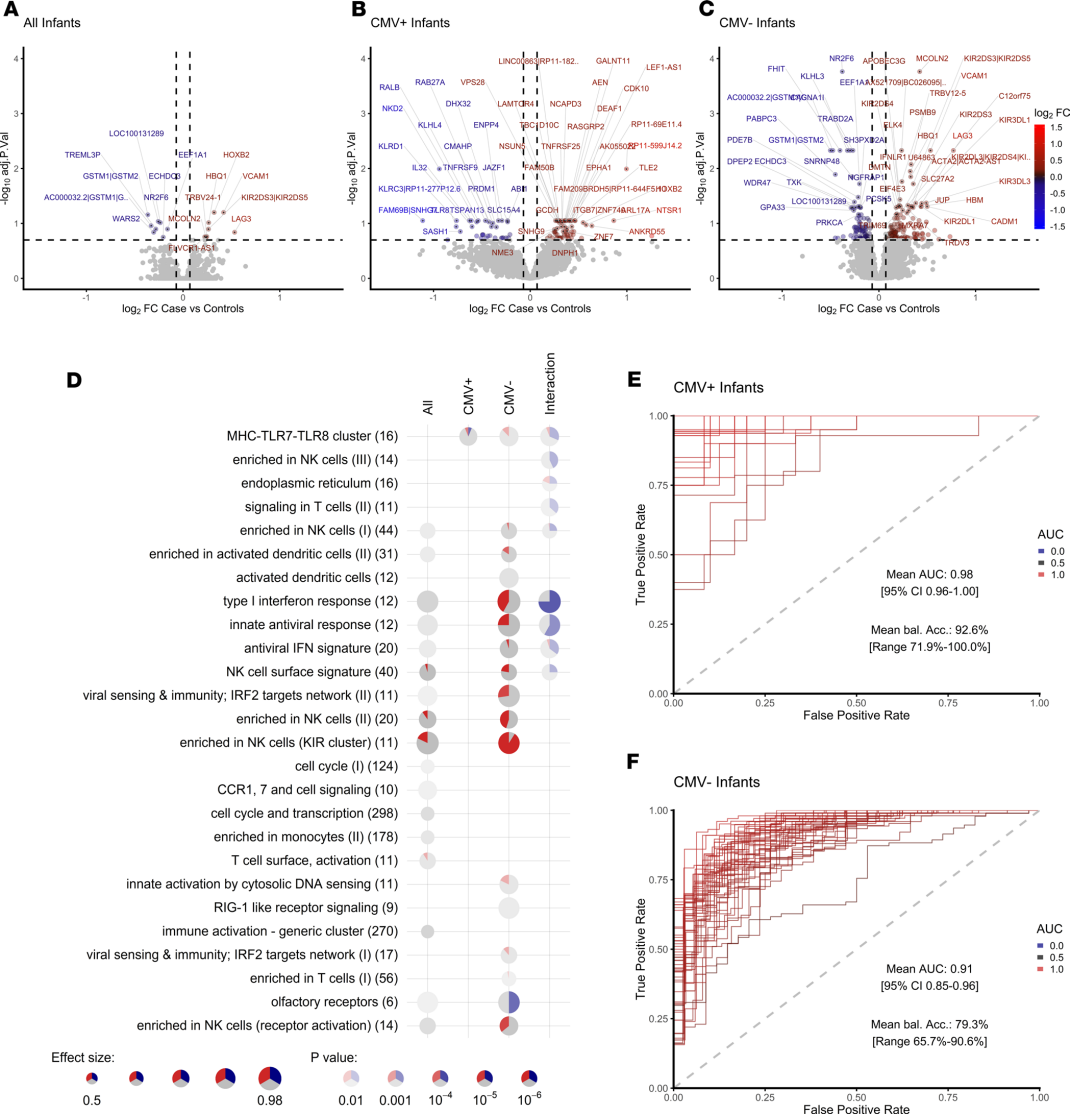
Transcriptomic correlates of risk of TB disease are different in CMV^+^ and CMV^–^ infants. Volcano plots showing magnitude and significance of differential expression between all case and control infants (**A**), CMV^+^ case and control infants (**B**), and CMV^–^ case and control infants (**C**). The top 50 significant genes are labeled, and horizontal and vertical dashed lines indicate 20% FDR and 5% change in gene expression, respectively. Log_2_ FC color code: blue, downregulated in cases vs. controls; gray, unchanged; red, upregulated in cases vs. controls. (**D**) Enriched modules for differential expression in case and control infants among all, CMV^+^, and CMV^–^ infants. Each row contains 1 module with the number of genes indicated. Each significantly enriched module at a *P* < 0.05 is shown as a pie chart. The size of the pie corresponds to the AUROC in the cerno test, and intensity of the color corresponds to the enrichment *q* value. The red and blue color indicates the amount of significantly up- and downregulated genes, respectively, and gray color indicates the remaining nonsignificant genes within the category. The interaction term evaluates the statistical difference between changes in CMV^+^ and CMV^–^ infants. (**E** and **F**) AUROC of the classification performance of the artificial neural network model, which was trained using approximately 70% of the data, and risk of TB was predicted on the withheld 30% of the data (Supplemental Figure 3A). The process was repeated 50 times with random splits into training and test set (bootstrapping), and the AUROC was recorded for each round for CMV^+^ (**E**) and CMV^–^ (**F**) infants.

Because CMV ELISpot–positive infants were at greater risk of disease, we stratified transcriptome data by CMV status to identify transcripts able to classify case and control infants. When CMV^+^ and CMV^–^ infants were analyzed together, 16 genes were significantly differentially expressed between cases and controls (Figure 4A and Supplemental Table 3). LAG3 and VCAM1, known markers of T cell activation (28, 32, 33), had the greatest fold increase in expression in case infants. To test our ability to classify infants into cases or controls, we split samples randomly into a 70% training set and 30% test set and trained an artificial neural network. The process was repeated 50 times with random splits into training and test set (bootstrapping), and area under the receiver operating characteristic (AUROC) and accuracy were recorded to assess predictive stability (Supplemental Figure 3A). When all infants were included in the analysis, we could classify infants into cases and controls with an average accuracy of 67% (AUROC, 0.77; 95% CI, 0.69–0.85; Supplemental Figure 6A). Prediction accuracies were comparable when alternative, widely used classification algorithms were used (Supplemental Figure 3B).

In CMV^+^ healthy infants who developed TB in the following 3 years (cases), the NK cell–associated cytokine IL-32 and the NK cell–specific lectin-like receptors KLRC1 and KLRC3 were among transcripts with the greatest decrease in fold-change of expression and the highest predictive power (Figure 4B, Supplemental Figure 4, and Supplemental Table 4). IL-32 enhances maturation of monocytes to macrophages and has been shown to be important for protection against *M.tb* (34). In our cellular analysis, we observed decreased frequencies of CD3^–^CD8^–^CD4^–^ (triple negative) CD16^–^ and CD16^+^ NK cells in infants who develop TB disease in the next 3 years when compared with CMV^+^ infants who do not develop disease (Supplemental Figure 6B).

In CMV^–^ case infants, elevated expression of T cell activation markers, including LAG3 and VCAM1; markers of a Type I/II IFN response, including IFIT3; and enhanced expression of a broad range of both activated and inhibitory KIR receptors, including KIR2DL1, KIR2DL3, KIR2DL4, KIR2DL5A, KIR2DS3, KIR2DS5, KIR3DL1, and KIR3DL3, were observed (Figure 4C and Supplemental Table 5). Modular pathway analysis showed enrichment for NK and KIR cluster genes and a Type I/II IFN antiviral immune response in CMV^–^ case infants when compared with controls (Figure 4D). However, we saw no evidence of increased NK cell frequency in infants who develop TB disease in our cellular analysis (Supplemental Figure 6B).

TB risk–associated transcripts and immune pathways were different among CMV^+^ and CMV^–^ infants, as highlighted by a modular pathway analysis, which uses an interaction term to compare pathways associated with TB risk among CMV^+^ and CMV^–^ infants (Figure 4D and Supplemental Table 6).

When infants were stratified by CMV status, we were able to classify cases and controls within the CMV^+^ cohort with 92% average balanced accuracy and within the CMV^–^ cohort with 89% average balanced accuracy, with an average AUROC of 0.98 (95% CI, 0.96–1) and 0.9 (95% CI, 0.85–0.96), respectively (Figure 4, E and F, and Supplemental Figure 5).

To validate the classifier signatures, we used raw data from an independent cohort of 10-week-old South African infants vaccinated with BCG at birth and unknown CMV status (NCBI’s Gene Expression Omnibus [GEO] database, GSE20716; ref. 5). Infants were enrolled into an efficacy trial of intradermal or percutaneous delivery of Japanese BCG at birth (3). A nested correlates of risk study was performed using blood from 10-week-old healthy infants who developed TB disease within the following 2 years (5, 35). We sought to remove technical differences between the 2 array data sets to enable validation of classifier signatures (Supplemental Figure 7A); however, we could not fully normalize expression between the 2 data sets, most likely due to the age difference between infants in the 2 cohorts (2–3 months in GSE20716 and 4–6 months in the MVA85A efficacy trial) (Supplemental Figure 7B). Despite these cohort differences and the loss of more than half of the genes in our signature due to microarray platform differences, we were able to predict risk of TB using our CMV^–^ classifier with an accuracy of 63.9% and an AUROC of 0.71 (95% CI, 0.63–0.79; Supplemental Figure 7C). Next, we attempted to improve accuracy by prediction and removal of infants with suspected CMV infection. The number of CMV^+^ infants with more than 1 predicted positive sample was low (*n* = 5, Supplemental Figure 7D) and excluding these infants led to a slight but not significant improvement in balanced accuracy of 65.6% and an AUROC of 0.74 (Supplemental Figure 7E). In all of the analyses, we saw greater numbers of differentially expressed genes when infants were stratified by CMV status. Many of the immune pathways associated with risk in CMV^–^ infants are in the opposite direction in CMV^+^ infants; therefore, these are canceled out when the 2 groups are combined.

Finally, we looked for enrichment of transcripts from the 16–peripheral blood gene correlate of risk (CoR) signature associated with progression to TB disease in *M.tb*–infected adolescents (36) among our CMV^+^ and CMV^–^ infants. This adolescent CoR signature was significantly enriched among case infants when compared with controls, and the enrichment was strongest among CMV^–^ infants (Supplemental Figure 8A). However, the adolescent CoR signature was not able to accurately classify infants into cases and controls (Supplemental Figure 8B). Including the CoR score in our infant network analysis, we show that the CoR score correlates with the frequency of inflammatory monocytes and with our CMV^–^ classifier signature (Supplemental Figure 8C). Our CMV^+^ classifier signature is positively correlated with T cell frequency and negatively correlated with monocyte and CD16^+^ NK cell frequency.

A network representation of positively correlating cell populations (Spearman’s rho, *P* < 0.05) revealed 3 major clusters with the adolescent CoR and infant CMV^–^ classifier signature clustering with inflammatory and classical monocytes (Supplemental Figure 8D).

These findings further support distinct immunological correlates of risk of TB disease in CMV^+^ and CMV^–^ infants. Taken together, our data show increased T cell activation, KIR receptor signaling, and Type I IFN response in CMV^–^ case infants and decreased NK cell–associated transcripts in CMV^+^ case infants when compared with their respective controls. Furthermore, we identified transcriptomic signatures, which can identify infants with risk of TB disease with high accuracy in CMV^+^ and CMV^–^ infants, and we were able to validate the CMV^–^ biomarker signature in an independent study with moderate accuracy.

### T cell activation associates with lower mycobacterial antigen–specific immune response following immunization with MVA85A and BCG.

**Figure 5:**
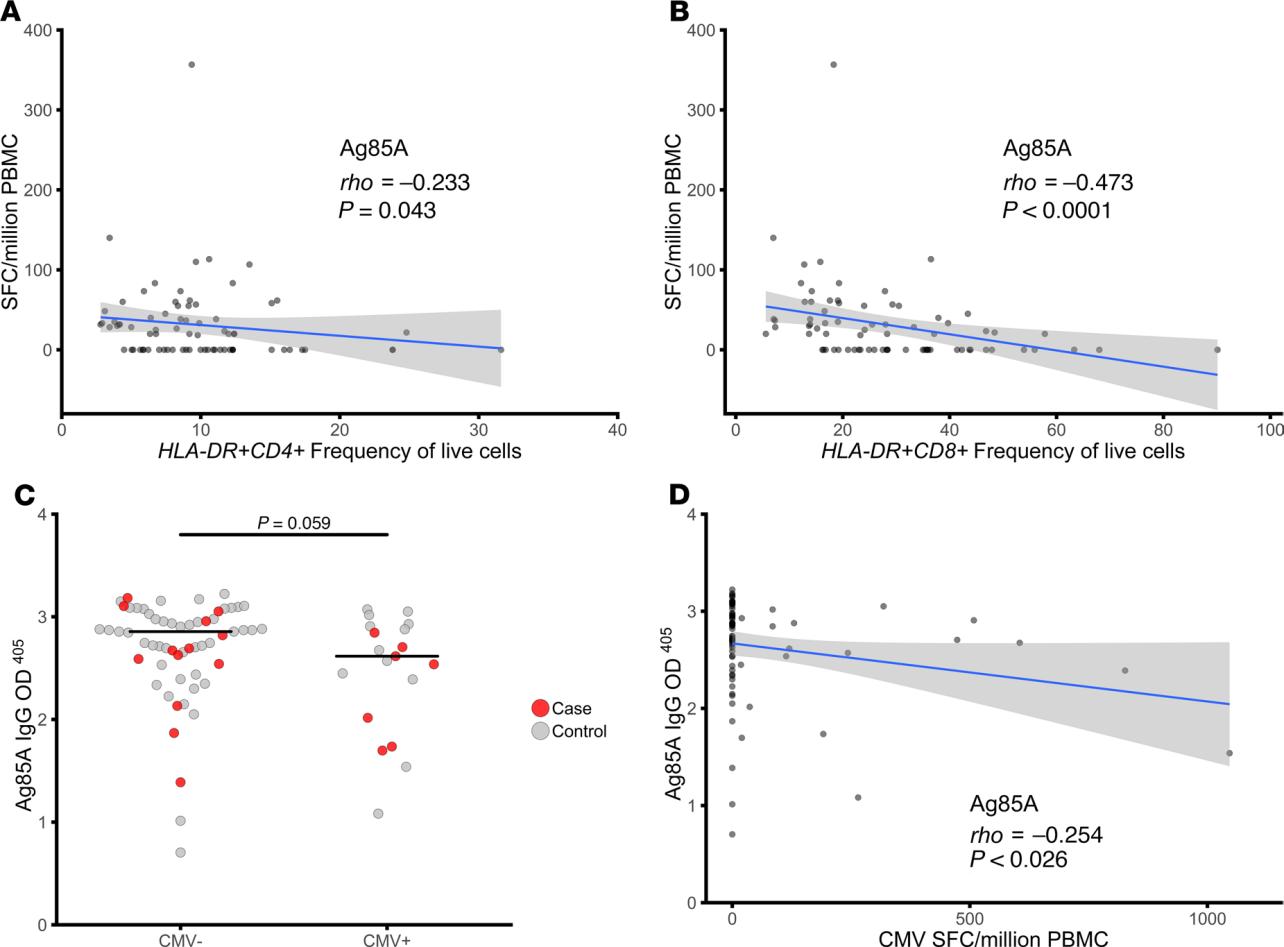
T cell activation is associated with lower mycobacterial antigen–specific immune response following immunization with MVA85A. (**A** and **B**) The D28 IFN-γ ELISpot response to Ag85A was inversely correlated with both activated CD4^+^ T cell (**A**) and activated CD8^+^ T cell (**B**) frequency. (**C**) There was a trend for lower anti–Ag85A IgG in CMV^+^ compared with CMV^–^ infants following immunization with MVA85A. (**D**) Anti–Ag85A IgG was inversely correlated with CMV ELISpot response. Mann Whitney *U* test and Spearman’s rho correlation. Red, cases; gray, controls.

To assess the impact of T cell activation and CMV infection on the ability to mount an antigen-specific response following immunization with MVA85A, we examined mycobacterial antigen–specific IFN-γ responses and anti–Ag85A IgG in MVA85A-immunized infants at D28 following immunization with MVA85A. IFN-γ responses to Ag85A, measured on D28, were inversely correlated with activated CD4^+^ and CD8^+^ T cell frequencies (Figure 5, A and B). There was a trend toward lower mycobacterial antigen–specific IFN-γ responses and lower anti–Ag85A IgG in CMV^+^ when compared with CMV^–^ infants (*P* = 0.058, Mann-Whitney *U* test), and anti–Ag85A IgG was inversely correlated with CMV ELISpot response in MVA85A immunized infants (Spearman’s rho, *P* = 0.026, Figure 5, C and D).

These data show that T cell activation and CMV infection influence MVA85A boosting of an antigen-specific immune response.

## Discussion

We demonstrate, in healthy infants, that CD8^+^ T cell activation and prior or subclinical infection with CMV (defined by a positive CMV-specific T cell response and treated as a binary variable) are associated with increased risk of developing TB disease over the next 3 years of life. We also show that CMV^+^ infants acquire TB disease earlier than CMV^–^ infants. These data complement our previous finding that CD4^+^ T cell activation was associated with TB disease risk in infants and adolescents in a community with high TB incidence (6). CMV has been implicated in the etiology of TB, supported by epidemiological associations between the 2 diseases (21–25). In Gambian infants, CMV infection induced profound CD8^+^ T cell differentiation and activation, which persisted up to 2 years after infection (37, 38). Consistent with this effect, we show that infants with a positive T cell response to CMV peptides have a transcriptional signature associated with CMV-specific CD8^+^ T cells (27). However, among CMV^+^ infants, T cell activation markers were not differentially expressed between case and control infants, most likely because CMV itself is a strong driver of T cell activation, thus confounding the signature within this group.

In CMV^+^ infants, transcripts associated with NK cells had lower expression, and NK cell frequency was lower in cases when compared with controls. A role for NK cells in protection from TB disease has been demonstrated both in humans and animal models (39–45). The CD94 and NKG2C/KLRC2 transcripts had among the greatest decreases in fold-change of expression and the highest predictive power in identifying TB cases among CMV^+^ infants. *KLRC2* deletion is associated with reduced numbers of mature NK cells and increased susceptibility to HIV infection, certain autoimmune conditions, and cancer (46–49). We hypothesize that increased susceptibility to TB disease in CMV^+^ infants may result from loss of control of CMV replication and/or impairment of NK cell function due to *KLRC2* gene deletions in some individuals (21).

Among CMV^–^ infants who went on to develop TB disease, we observed upregulation of transcripts associated with T cell activation, including LAG3 (28), which is induced during active TB in a nonhuman primate model (50). We also found multiple transcripts and pathways that are typically altered during viral infection among CMV^–^ infants. This may be due to underestimation of the prevalence of viral infection, as we used only IFN-γ responsiveness to EBV and CMV CD8+ T cell epitopes as a measure of viral infection. Detection of viral DNA would allow more accurate diagnosis of viral infection; however, this was not possible due to the very limited samples collected from these infants. Moreover, viruses or bacteria other than those measured in this study (i.e., CMV and EBV) may be contributing to risk of TB disease among these infants. Expression of a broad range of both activating and inhibitory KIR receptor transcripts was elevated in CMV^–^ case infants. Exposure to multiple viral infections drives high diversity of KIR expression and lowers the availability of naive NK cells to respond to future infectious challenge, resulting in susceptibility to HIV (51, 52). We were able to identify different classifier signatures among CMV^+^ and CMV^–^ infants and were able to verify our CMV^–^ signature in an independent cohort of infants (5). In the independent cohort, infants were younger (2–3 months of age), and few infants were classified as CMV^+^. CMV infection and viral replication is low at birth, peaks at 3–6 months of age, and declines to plateau at 8–10 months of age (53). At 4–6 months of age, infants recruited in to the MVA85A efficacy trial (4) were at the peak age of CMV viral replication in infancy.

Our transcriptional evidence of immune activation in infants who develop TB disease is consistent with the observations of Zak et al. (36), who reported increased expression of Type I/II IFN–associated transcripts in *M.tb*–infected adolescents who progressed to TB disease. Increased Type I/II IFN transcripts have also been observed in patients with active TB disease, when compared with *M.tb*–infected or –uninfected controls (54–58). Recent data from the *M.tb*–infected adolescent study has shown that an increased Type I/II IFN response precedes a shift toward an elevated monocyte/lymphocyte ratio and an increase in T cell activation, which are detected closer to the time of TB disease (59). The authors suggest that the initial elevation in Type I/II IFN could be driven by viral infection and that this could then trigger the immune events that lead to TB susceptibility (59). Consistent with this, we found a correlation of the adolescent CoR score with an EBV-specific T cell response and with elevated CD14^+^CD16^+^ inflammatory cells. We also observed significant enrichment of the 16-gene CoR signature identified by Zak et al. among our CMV^–^ case infants (36). However, the CoR signature was not able to accurately classify case and control infants. In our study, the infants were not infected with *M.tb*, had no symptoms of TB disease, and had no known exposure to TB in the household during enrollment or when the blood samples were obtained. In addition, since incident TB disease was diagnosed months to years after blood sample collection, it is unlikely that the elevated Type I/II IFN–associated transcriptomic signatures we observed are a result of subclinical TB disease.

One limitation of this study is the small number of CMV^+^ infants (*n* = 18); the potential association between CMV infection and risk of TB disease now requires validation in a larger cohort with adjustment for known confounders, such as TB exposure and socioeconomic status, although socioeconomic status is known to be largely homogeneous in this particular population in the Western Cape (60). Samples were not available for serological analysis; therefore, CMV-specific T cell responses were measured as an indicator of CMV infection. However, CMV-specific IFN-γ responses have been shown to correlate with antibody responses (61, 62), and CMV-ELISpot and QuantiFERON assays are available and perform well as diagnostic tests for CMV infection and CMV DNA viremia (63). Furthermore, using cellular immunity as a marker of infection allows differentiation of young children with prior exposure to CMV from those with passive maternal antibody (62, 64). Although consistent with the etiology of CMV infection in infants, we are unable to confirm that immune activation was chronic, as only 2 samples were collected 4 weeks apart, and it is possible that T cell activation could have been driven by acute infection in some cases. While only CMV and EBV were measured in this study, other infections may also contribute to T cell activation, and CMV may be acquired or activated as a result of comorbidities, including respiratory or diarrheal infections. However, blood sampling at time of enrollment and a stringent screening process ensured that any infant with sign or symptom of coinfection was excluded from the study. Safety bloods and frequent follow-up showed no clinical indication of active infection.

All infants in this study received BCG at birth, and the T cell response to BCG peaks at 2–3 months of age (65). Viral infections during the development of the BCG-specific immune response may impair the development of protective immunity, as has been suggested by studies in Malawi (66). In the Gambia, exposure to HIV in utero and being born in the wet season, associated with increased respiratory and diarrheal disease, have been shown to impact the BCG antigen–specific T cell response following vaccination (37, 67). We observed an inverse correlation between CD4^+^ and CD8^+^ T cell activation and antigen-specific T cell responses following immunization with MVA85A, suggesting that T cell activation may be associated with decreased vaccine boosting in infants. This confirms previous observations where increased immune activation was associated with lower immune responses to MVA85A in both infants and adults (68, 69). It could also explain why immune responses to MVA85A were lower when administered within a week of vaccination with DTwP-Hib and hepatitis B in the Expanded Programme on Immunization (EPI) schedule (70).

Childhood TB is difficult to diagnose and treat, and improved strategies are needed to control TB in children (71). We found that a CMV-specific IFN-γ response was associated with increased risk of developing TB disease in infants and that distinct immune pathways were associated with TB disease risk in CMV^+^ and CMV^–^ infants. We suggest that viral infection may increase the risk of progression to TB disease and may compromise the immune response to TB vaccines given in infancy. Strategies to reduce chronic viral infections such as the use of vaccines or antivirals, and protective behaviors including handwashing and glove use, are particularly important during pregnancy to reduce to frequency of congenital infection (72–75). Such interventions may have broader benefits in enhancing TB vaccine efficacy, reducing TB risk, and helping to reduce the global burden of childhood TB.

## Methods

### Case-control design.

Infants who were enrolled in an efficacy trial of the candidate TB vaccine MVA85A were included in this study (ClinicalTrials.gov, NCT00953927; ref. 4) (Figure 1). All infants received BCG within 7 days of birth. Infants were screened at 4–6 months of age, and only those with clinical parameters within the normal range who were HIV- and *M.tb*–uninfected infants and without known TB exposure were included in the trial. CMV and EBV analysis was conducted on a blood sample taken at this time point. Infants were randomized at 16–24 weeks of age to receive a single intradermal dose of MVA85A or placebo (Candin, a candida skin test antigen; AllerMed) (4). Transcriptomic analysis was performed using PBMC from infants who were included in our previously described case-control study (6). Briefly, infants who met the primary case definition for TB disease (Supplemental Table 7) were included as cases. Infants developed TB disease at a mean of 15 months (1–32 months) after baseline blood draw. For each case, 3 infants were randomly selected from a pool of controls (Figure 1). Infants were included in the control pool if they did not demonstrate *M.tb* infection as defined by a positive QuantiFERON TB Gold In-tube test (Cellestis); had not received TB treatment; and had not received isozianid preventive therapy during study follow-up. Matching was based on sex, ethnic group, CDC weight-for-age percentile (± 10 points), and time on study (± 9 months).

Infant PBMC samples collected from 2 time points, D0 and D28, were combined for testing of both cellular parameters and transcriptional signatures associated with risk of TB disease. Only infants for whom a sample was available at both D0 and D28 were included in the analysis, resulting in a combined analysis of a maximum of 98 samples from 49 cases and 258 samples from 129 controls (actual numbers in analysis vary per availability of assay data and are listed in Table 1 and Figure 1).

The demographic and clinical characteristics of CMV^+^ vs. CMV^–^ infants are summarized in Supplemental Table 8 and were similar between groups. Socioeconomic status is known to be largely homogeneous in this particular population in the Western Cape.

### Cell culture.

PBMC were retrieved from liquid nitrogen storage, thawed, and rested for 2 hours in media containing DNase (Merck Chemicals Ltd.) to aid the removal of debris from dead and dying cells. After 2 hours, cells were counted and immediately transferred to cell culture plates for stimulation for RNA extraction, ELISpot assays, or measurement of cell-surface markers assessed by flow cytometry. Viability of thawed PBMC was assessed using flow cytometry with Live/Dead Violet stain (Invitrogen). Phytohemagglutinin (PHA) was included as a positive control for cell viability on ELISpot plates.

### RNA processing.

Rested PBMC (1 × 10^6^) were resuspended in single wells of 200 μL RPMI (Sigma) supplemented with 10% FBS (Biosera) and L-glutamine (Sigma). Cells were stimulated for 12 hours at 37°C in single wells containing live BCG solvent for suspension for injection (SSI) from pooled vaccine vials (approximately 2 × 10^5^ CFU/mL). After 12 hours of stimulation, cells were pelleted and lysed in RNA lysis buffer (RLT, Qiagen). RNA was extracted using the RNeasy Mini Kit (Qiagen) per the manufacturer’s instructions with the following modification; in the first step, an equal volume of 80% ethanol was added to cells lysed in RLT buffer, mixed, and total volume transferred to an RNeasy column. RNA was quantified by NanoDrop (Thermo Fisher Scientific) and stored at –80°C until use. Extracted RNA was amplified and labeled with biotin using the Illumina Total Prep kit (Ambion) per manufacturer’s instructions. Amplified RNA was assessed by NanoDrop and Bioanalyzer for quantity and quality prior to hybridization. Hybridization to Illumina HT-12 arrays was performed per manufacturer’s instructions. Arrays were scanned using an Illumina iScan machine, and data were extracted using Genome Studio software.

*Ex vivo IFN-γELISpot assay*. The ex vivo IFN-γ ELISpot assay was performed as previously described using a human IFN-γ ELISpot kit (capture mAb 1-D1K) (Mabtech) (6). Briefly, duplicate wells containing 3 × 10^5^ PBMC were stimulated for 18 hours with antigen, PHA (Sigma), or media alone. Antigens included a single pool of Ag85A peptides (2 μg/mL/peptide; Peptide Protein Research); BCG (2 × 10^5^ CFU/mL; Statens Serum Institute); purified protein derivative (PPD) from *M*.*tb* (20 μg/mL; Statens Serum Institute); and peptide pools containing known CD8^+^ T cell epitopes from EBV (15 peptides) and CMV (5 peptides) (2 μg/mL/peptide, AnaSPEC). Results are reported as SFC per million PBMC, calculated by subtracting the mean of the unstimulated wells from the mean of antigen wells and correcting for the number of PBMC. A response was considered positive if the mean number of spots in the antigen well was at least twice the mean of the unstimulated wells and at least 5 spots greater.

### Cell surface flow cytometry.

As previously described (6), PBMC were washed and stained with 5 μL Live/Dead Violet (Invitrogen) followed by surface staining with the following titrated antibodies: 0.5 μL CD3-AF700 (clone UCHT1, eBioscience), 2 μL CD4-APC (clone RPA-T4, BioLegend), 2 μL CD8-eFluor605 (clone RPA-T8, eBioscience), 2 μL CD14-PE/Cy7 (clone HCD14, BioLegend), 2 μL CD16-AF488 (clone 3G8, BioLegend), 1 μL CD19-PE/Cy5 (clone HIB19, BioLegend), 2 μL CD25-APC/Cy7 (clone BC96, BioLegend), 2 μL CD127-NC650 (clone eBioRDR5, eBioscience), and 15 μL HLA-DR–PE (clone L243, BioLegend). Fluorescence minus one (FMO) controls were used to set gates for CD25, CD127, and HLA-DR. Samples were acquired on a BD LSR II flow cytometer. Results are presented as percentages of cells after excluding dead cells and doublets. CD4^+^ and CD8^+^ T cells were identified as CD3^+^ cells, while CD14^+/−^ and CD16^+/−^ cells were identified as CD3^−^ and CD19^−^ populations. CD25^+^CD27^−^ populations were gated on the CD4^+^ cells. The network representation of cell populations positively correlated among all infants was done using the igraph package in R. To identify closely related clusters (communities) within the network, the cluster_optimal function was used, implementing an algorithm described in Brandes et*.* al*.*(26).

### Transcriptional analysis.

Raw, probe level summary values exported from Illumina GenomeStudio 2011 of Illumina HumanHT 12 V4 microarrays were imported into R using BeadArray (76). Probes were background corrected using negative control probes followed by quantile normalization using the neqc command (77). The analysis was restricted to probes with a detection *P* < 0.01 in at least 10% of the samples and probes matching to the transcript definition of the following databases (in descending importance) with at most 2 mismatches, no insertions, and a minimum mapping length of 40 bases: GENCODE version 23, RefSeq (refMrna.fa), and GenBank (mrna.fa) downloaded in August 2015 from http://hgdownload.cse.ucsc.edu/goldenPath/hg38/bigZips/

A linear model was fitted using limma (78) to determine differential expression adjusted for vaccine, day, stimulus, sex, age, ethnicity, and batch effects. Array quality weights were incorporated (79) to account for between-array quality differences. To account for between-patient correlations, the duplicateCorrelation command from the limma package was used. Nominal *P* values were corrected for multiple hypotheses testing using the Benjamini-Hochberg procedure (80). Due to the heterogeneity of the samples, a lenient cut off at an FDR of 20% was chosen to identify genes as significantly differentially expressed. In total, 221, 101, and 16 probes mapping to 203, 95, and 16 genes were significantly differentially expressed between case and control infants within CMV^–^, CMV^+^, and the combined group, respectively. For the comparison of EBV^+^ vs. EBV^–^, CMV^+^ vs. CMV^–^, and CMV–strongly positive (ELISpot > 100 SFC/million) vs. CMV^–^, in total, 334, 14, and 103 probes mapping to 296, 14, and 97 genes were significantly differentially expressed, respectively.

Pathway analysis, performed using gene set enrichment analysis (GSEA), was carried out using the cerno test from the tmod package in R. Modules for the enrichment analysis were taken from Li et al. (81). Data sets used for comparative analysis to CMV-infected CD8^+^ T cells were obtained from GEO by downloading GSE12589 and GSE24151.

### Classification.

For classification into cases and control samples, we used normalized microarray intensities adjusted for scan date, sample collection time point (D0 or D28), and stimulation (unstimulated or BCG stimulated). Features for training were selected by using the significant probes at 20% FDR and selecting only the probe with the highest average expression per gene, giving the final transcriptional signature size of 95, 203, and 16 genes for CMV^+^, CMV^–^, and all infants, respectively. Each infant was represented either by sets of 2 samples (unstimulated and BCG stimulated either at D0 or D28) or by sets of 4 samples (unstimulated or BCG stimulated at D0 and D28). To avoid overfitting, we implemented a modified nested cross-validation scheme such that only complete sample sets per infant were assigned to either test or training set at each splitting iteration during the cross-validation process.

Model training was performed using a neural network model as implemented in the nnet package through the caret interface in R (82). In the outer loop, samples were split 50 times with replacement into training (about 70%) and test sets (about 30%) to evaluate model performance and feature importance. For model parameter tuning in the inner loop, each training set was split into training and validation sets using a leave-1-infant-out cross validation scheme, and the AUROC was recorded as performance metric over a grid of 4 size and 4 decay parameter combinations. All accuracies stated in the manuscript are provided as balanced accuracies (83) to account for the imbalance of the case and control infants within our cohort.

To validate our classification results, an independent cohort of 10-week-old South African infants vaccinated with BCG at birth was obtained as raw expression data from GEO by downloading GSE20716. Study batch was removed with the ComBat command in R using parametric adjustment, risk of TB as null model, and the validation cohort as reference in order to avoid any bias on the validation cohort. For prediction of risk of TB, 112, 50, and 5 probes overlapped between Illumina HumanHT 12 V4 and Illumina HumanRef-8 V2 for within CMV^–^, CMV^+^, and the combined group, respectively. CMV status prediction was performed using 55 overlapping probes, which were differentially expressed between CMV^–^ and CMV–strongly positive (ELISpot > 100 SFC/million) infants at an FDR of 20%. Only infants with at least 2 positive samples were labeled as suspected CMV^+^.

### Data availability.

Raw and normalized expression data have been deposited at GEO under the accession number GSE98550.

### Statistics.

Data were analyzed using R. CLR was conducted to identify associations between cellular immune parameters or CMV and/or EBV IFN-γ ELISpot responses and risk of TB disease. Correlations between 2 measures were performed using Spearman’s rho. A log-rank Mantel-Cox test was used to determine whether infants with a positive CMV and/or EBV IFN-γ ELISpot response developed TB earlier than negative infants.

### Study approval.

Samples used in this study were taken from an efficacy trial of the candidate TB vaccine MVA85A (ClinicalTrials.gov, NCT00953927; ref. 4) (Figure 1). This trial was approved by the University of Cape Town Faculty of Health Sciences Human Research Ethics Committee, Oxford University Tropical Research Ethics Committee, and the Medicines Control Council of South Africa.

## Author contributions

HAF, MAS, BL, SKM, MH, GH, HM, MT, JBM, TGE, WAH, TJS, and HM designed the research study; RT, MM, IS, SAH, MKO, LS, LM, AC, RHK, ZRMT, VN, and ES conducted experiments and acquired data; JM and HAF analyzed data; HAF, JM, and RT wrote the manuscript; and all authors provided assistance and critical review.

## Supplementary Material

Supplemental data

Supplemental Tables 1-6

## Figures and Tables

**Table 1:**
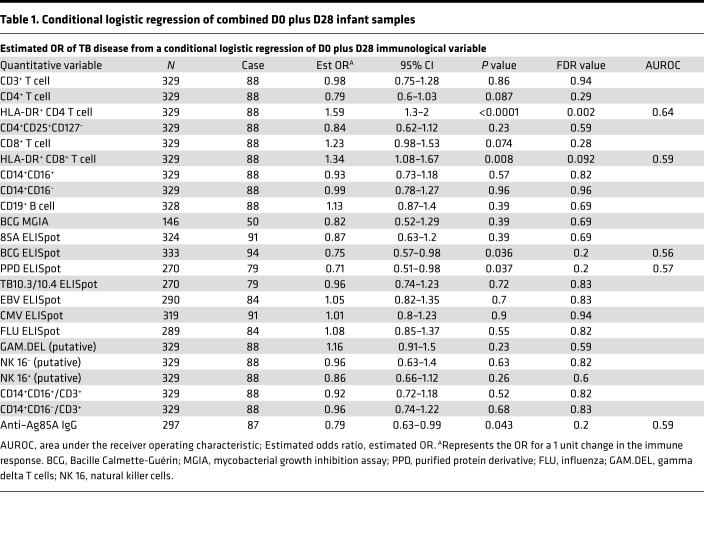
Conditional logistic regression of combined D0 plus D28 infant samples

## Version 1 11/07/2019

In-Press Preview

## Version 2 12/05/2019

Electronic publication
