# Supplementary material for: Cytomegalovirus infection is a risk factor for tuberculosis disease in infants
Source: JCI Insight. 2019 Dec 5;4(23):e130090. doi: 10.1172/jci.insight.130090 (PMC6962026; doi:10.1172/jci.insight.130090)
Supplement: Supplemental data [file jciinsight-4-130090-s197.pdf]

Any of the following criteria:

Isolation of *Mycobacterium tuberculosis* from any site.

Identification *of M tuberculosis* by an approved molecular diagnostic technique from any site.

Histopathology diagnostic for tuberculosis disease (eg, caseating granulomas).

Choroidal tubercle diagnosed by an ophthalmologist.

Miliary pattern on chest radiograph in an HIV-negative infant.

Clinical diagnosis of tuberculous meningitis (cerebrospinal fluid protein concentrations >0·6 g/L and pleocytosis of >50 cells per µL with >50% mononuclear cells) with features of basal meningeal enhancement and hydrocephalus on head CT.

Vertebral spondylosis.

One smear or histology specimen positive for auramine-positive bacilli from a normally sterile body site.

One of each of the following:

Evidence of mycobacterial infection defined as two acid-fast positive smears (each from a separate collection) that were morphologically consistent with mycobacteria from either sputum or gastric aspirate that were not found to be non-tuberculous mycobacteria bacteria on culture; QuantiFERON-TB Gold In-tube test conversion from negative to positive; or tuberculin skin test ≥15 mm.

Radiographic findings compatible with tuberculosis defined as ≥1 of the following factors identified independently by at least two of three paediatric radiologists serving on a masked review panel: calcified Ghon focus, pulmonary cavity, hilar or mediastinal adenopathy, pleural effusion, or airspace opacification.

Clinical manifestations compatible with tuberculosis defined as cough without improvement for >2 weeks; weight loss of >10% of bodyweight for >2 months; or failure to thrive, defined as crossing >1 complete major centile band (<97th–90th, <90th–75th, <75th–50th, <50th–25th, <25th–10th, and <10th–3rd weight-for-age centiles) downward for >2 months.

Supplementary Table 7. **Definition of endpoint 1.**

	**CMV+**<br>n=32	**CMV-**<br>n=140
Age, days	147 (13.7)	149 (14.3)
Sex, male	19 (50%)	67 (48%)
Ethnic group		
Black	3 (9%)	19 (14%)
Mixed race	29 (91%)	121 (86%)
CDCPWT	29.5 (28.1)	30.9 (27.9)

Supplementary Table 8. **Demographics and baseline characteristics of CMV + and CMV- infants included in the analysis**. Data are mean (SD) or n (%).

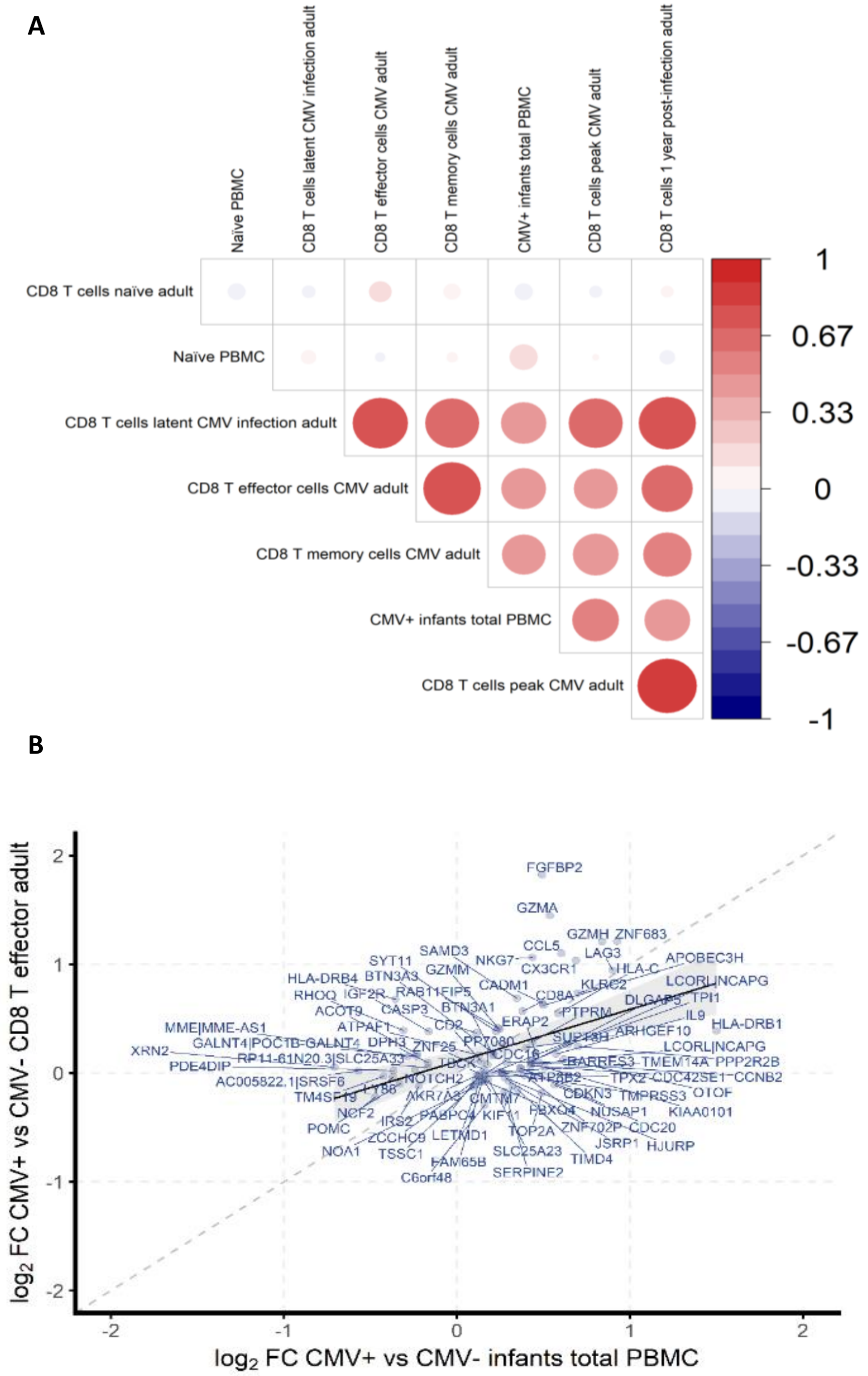

Supplementary Figure 1. **Prominence of CD8 T cell specific transcripts from total PBMC of CMV ELISpot positive infants.** A) Spearman's rho from correlations of gene expression from total PBMC of infants with a strong CMV ELISpot response >100 SFC/million with CD8+ T cells isolated from adults who were naïve or infected with CMV (transcripts with <20% false discovery rate (FDR)). B) Scatter plot of 84 overlapping transcripts between PBMC of infants with a strong CMV ELISpot response >100 SFC/million and transcripts from CMV virus specific effector CD8 T cells from CMV infected adults (GSE24151).

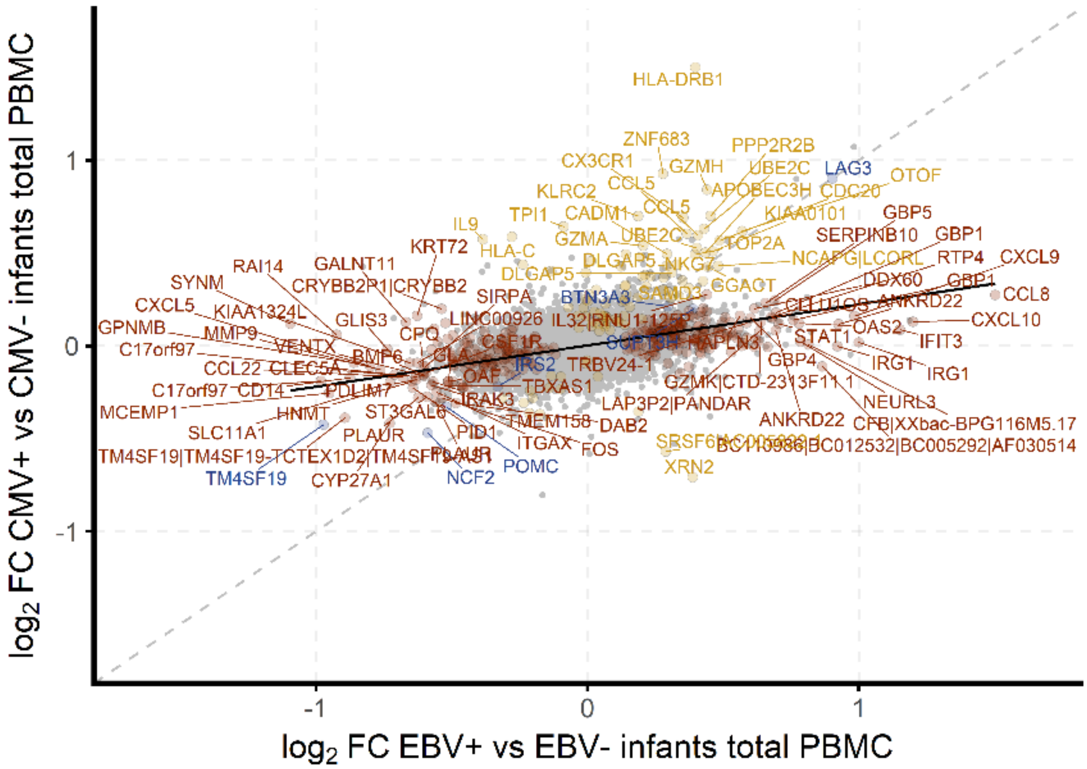

Supplementary Figure 2. **Little overlap in expression in infants who are either EBV or CMV ELISpot positive**. Scatterplot of fold changes in response to EBV infection (x-axis) plotted against changes in response to CMV infection (y axis). The almost horizontal linear regression line indicates stronger changes in response to EBV. At a false discovery rate (FDR) of 20%, seven genes are significantly differentially expressed in both comparisons.

A

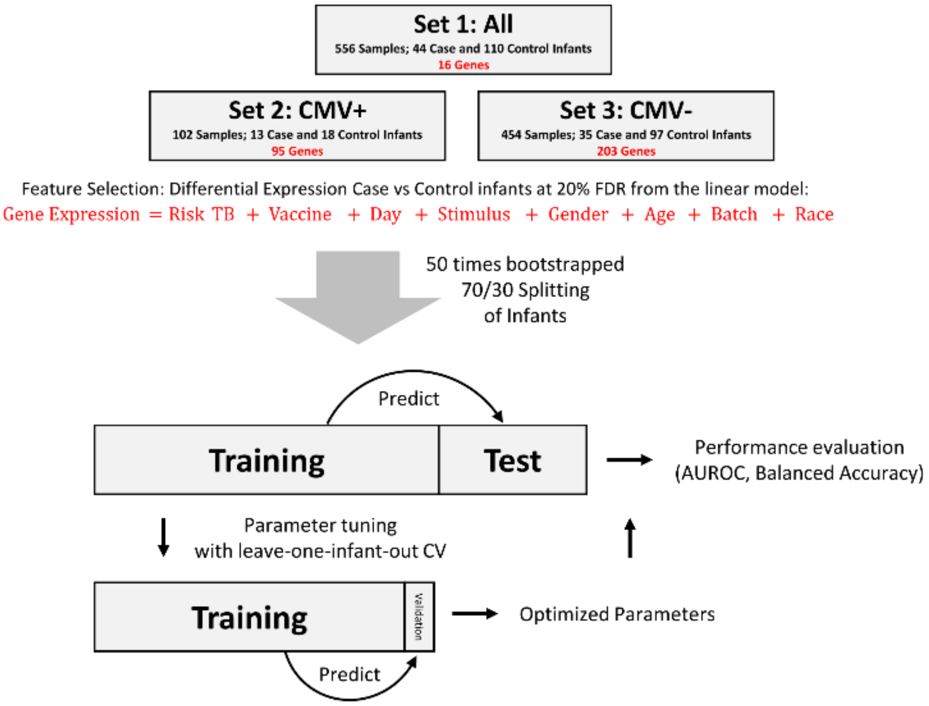

B

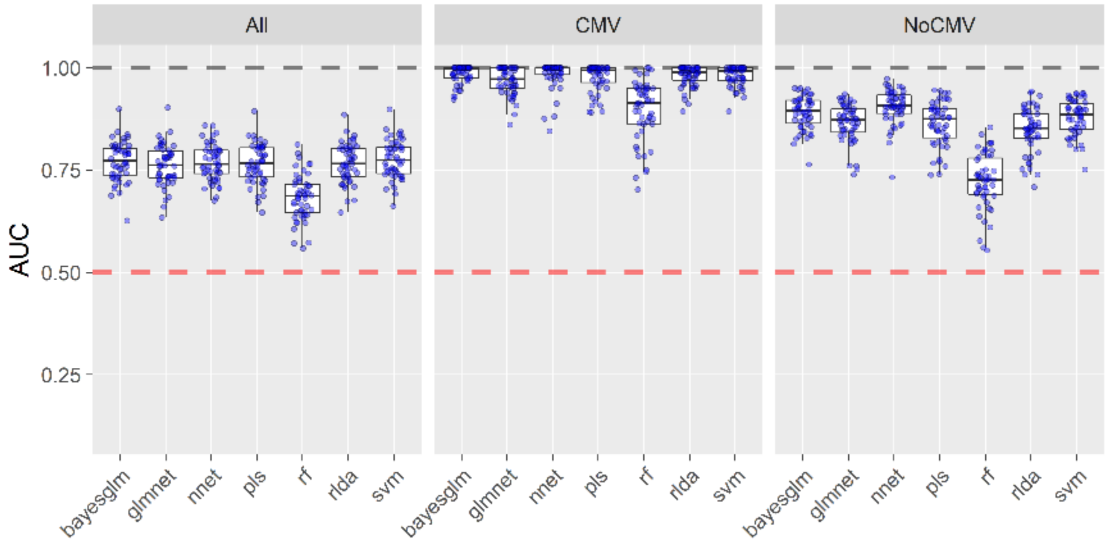

Supplementary Figure 3. **Classification strategy and model comparison** A) Overview of the classification strategy for TB risk prediction. An artificial neural network model was trained using approximately 70% of the data and TB risk was predicted on the withheld 30% of the data. Only significantly differentially expressed genes were used as features for classification for each set of infants and model parameters were tuned using leave one infant out cross validation. The process was repeated fifty times with random splits into training and test set (bootstrapping) and AUROC and balanced accuracy were recorded for each round. B) Classification performance comparison of seven models applied to the datasets. Full names of models and parameter tuning as follows: bayesglm: Bayesian Generalized Linear Model, no tuning parameters; glmnet: a grid of 4 lambda values as determined by a call to glmnet and a fixed alpha at 0.1 to enforce moderate feature selection; nnet: Neural Networks, grid of two unit size (1 and 3) and 3 decay (0.1, 1 and 2) parameters.; pls: Partial

Least Squares, grid of 10 components; rf: random forrest, grid of 20 mtry values; rlda: Regularized Linear Discriminant Analysis with Schafer-Strimmer estimator, no tuning parameters; svm: Support Vector Machine with linear kernel, grid of 10 C values.

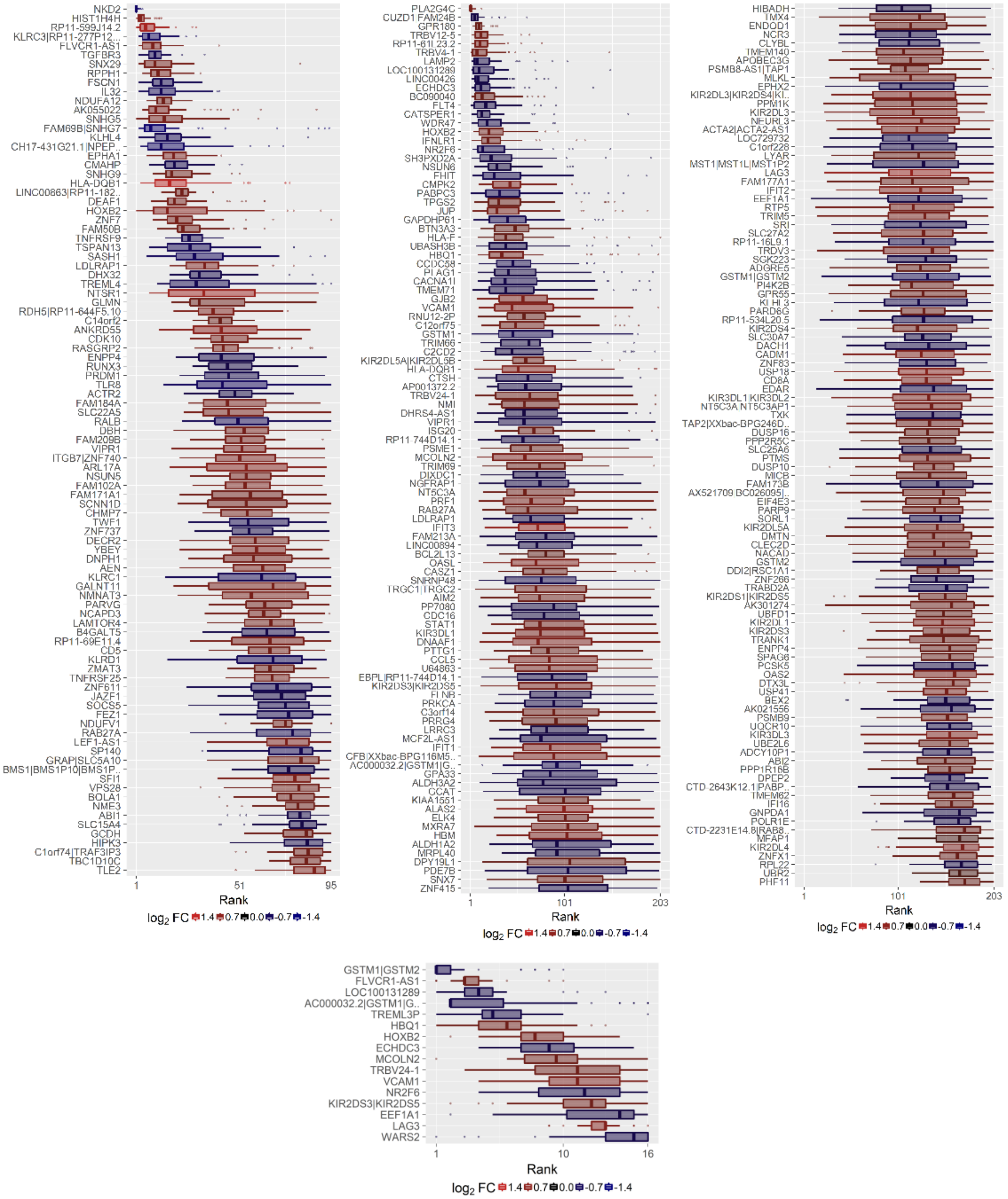

Supplementary Figure 4. **Boxplots showing the relative importance of features used to train the classifier for each data set.** For each repeat, features were ranked by variable importance and the relative rank was recorded. The lower the average number, the more often a feature has been assigned a high importance by the classifier during the fifty repeated predictions. The color indicates the Case vs Control infants log2 fold change estimate based on the differential expression analysis.

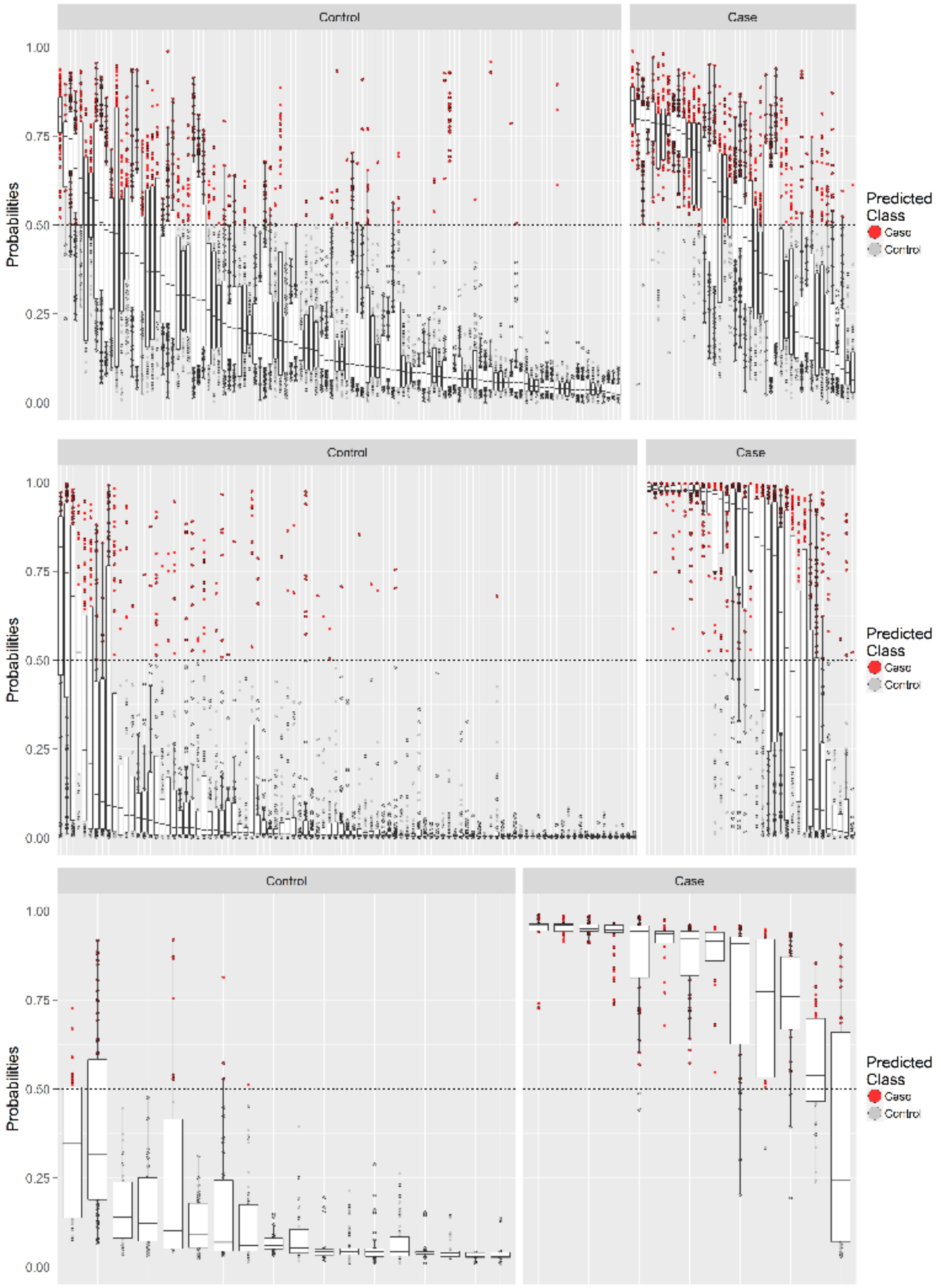

Supplementary Figure 5. **Average prediction accuracies for risk of TB are very high in infants stratified by their CMV status**. Box plots of probabilities assigned to each sample by the trained neural network. Each box plot represents one infant and contains stimulated as well as unstimulated samples and the results of fifty bootstrapped repeats of the prediction. Left and right panel divide the infants into cases and controls and point color indicates the classification decision by the model.

A

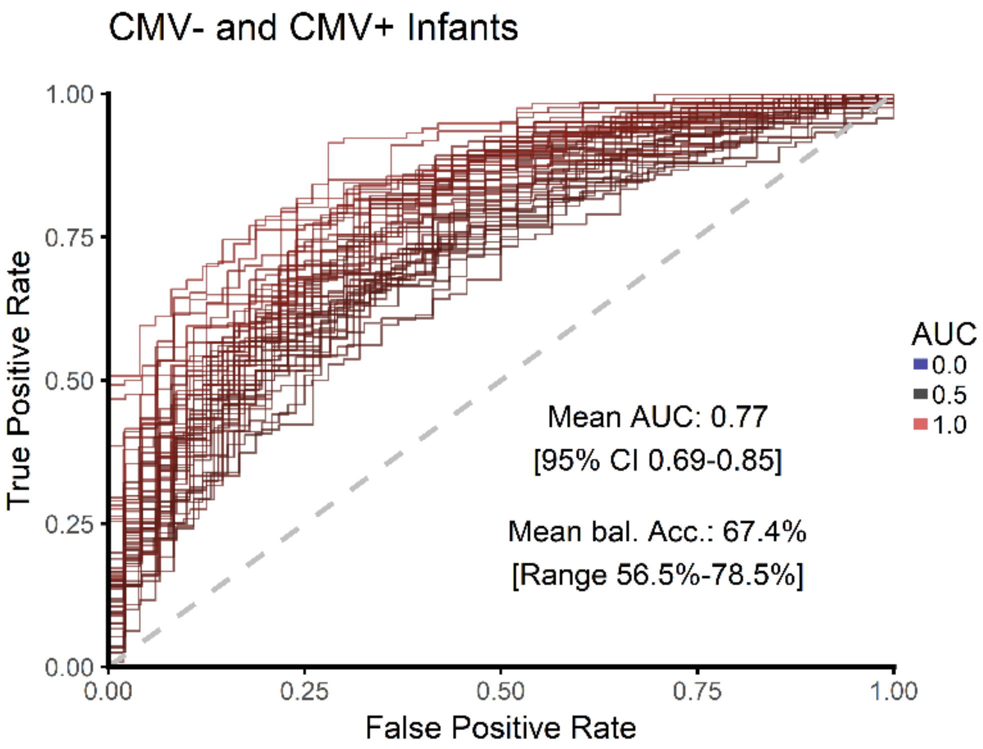

B

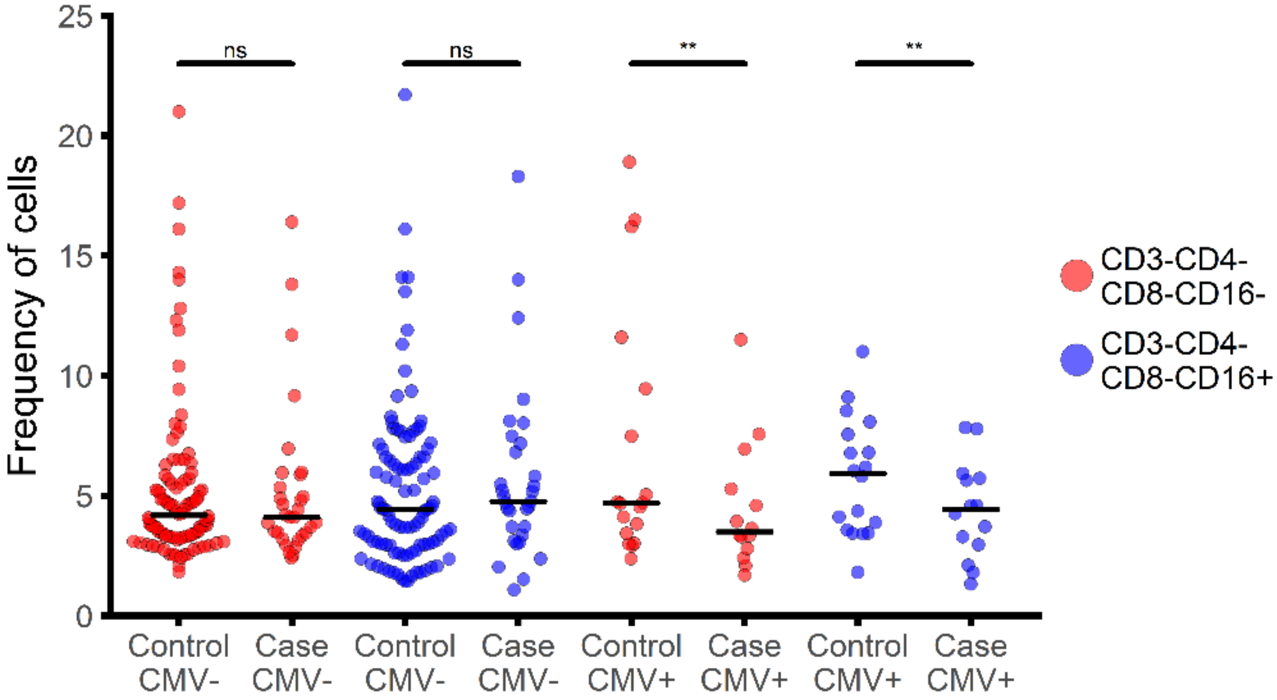

Supplementary Figure 6. **Classification into cases and controls using all samples.** A) Classification performance of the artificial neural network using CMV+ and CMV- infants. B) CD16- and CD16+ (putative) NK cell frequencies among case and control infants with and without a CMV response.

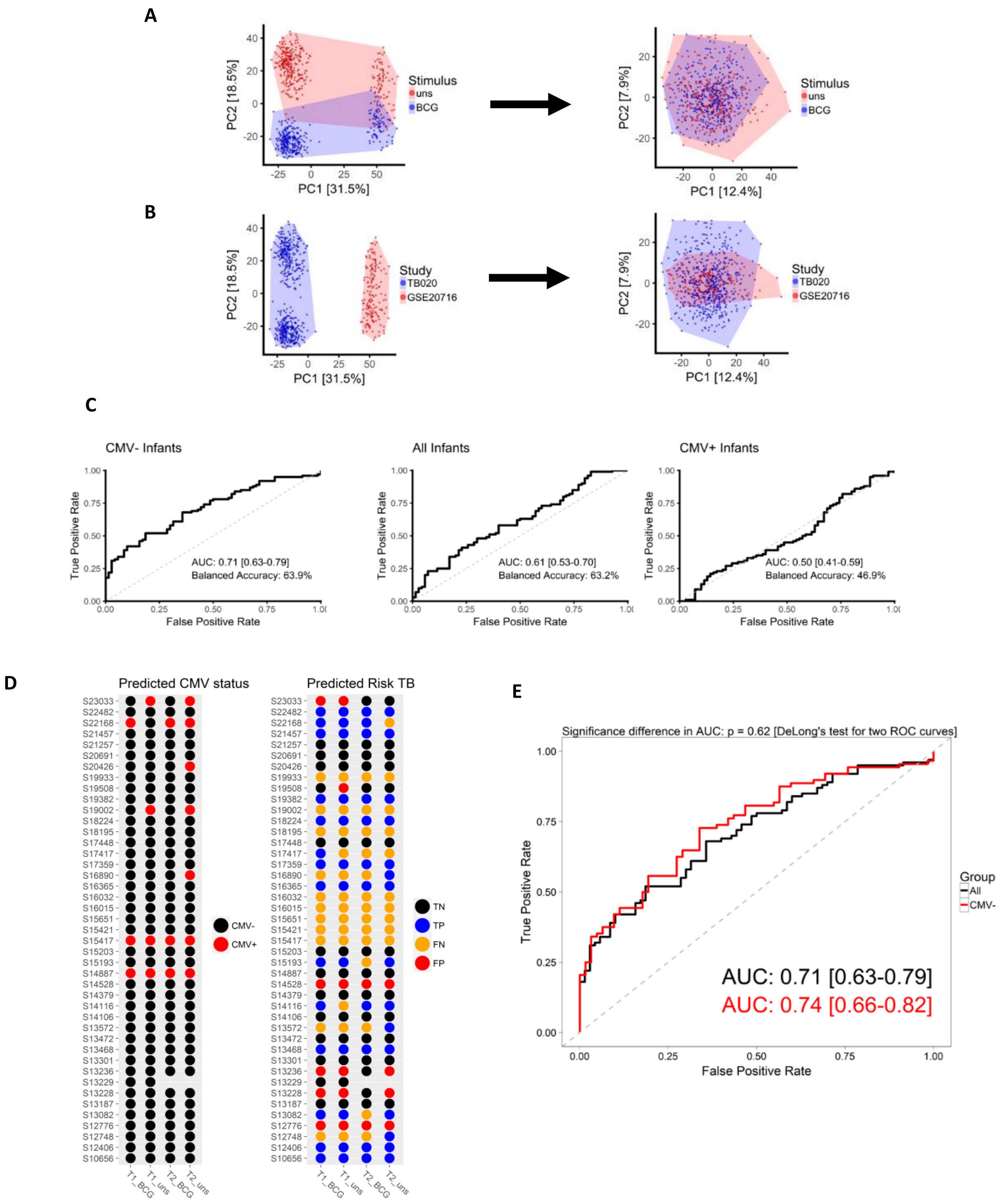

Supplementary Figure 7. **Validation of TB predictive biomarkers from CMV- infants in an independent cohort of BCG vaccinated infants** Raw data and detection p values from an independent cohort of 10 week old South African infants vaccinated with BCG at birth (GEO

gene set GSE20716(5)) were used as a validation cohort for the classifier signatures from CMV+ and CMV- infants. 20589 Probes from 182 samples were mapped from Illumina HumanRef-seq 8 arrays to Gencode v.23 and 8 outlier samples were excluded A) The effects of time point (4 or 12 hours) and BCG Stimulation (stimulated or unstimulated) were removed and 9195 probes identified as overlapping between the two studies. B) We were only partially able to remove differences in gene expression due to study of origin. The differences observed in PC2 are driven by age, which was non-overlapping in the two studies, 2-3 months in GSE20716 and 4-6 months in the MVA85A efficacy trial. C) Using 55 genes differentially expressed between infants with very high or low CMV titres at a false discovery rate (FDR) of 5%, 5 infants were identified to have two or more CMV positive samples. D) The charts represents one dot per sample and red colour indicates samples classified as CMV+. The TB Risk dot plot shows samples with correctly predicted Case (TP) and Control (TN) status as well as samples with wrong Case (FP) and Control (FN) labels. E). Excluding all five suspected CMV+ infants we were able to improve risk of TB prediction accuracyon GSE20716(5)slightly, with balanced accuracy of 65.6%.

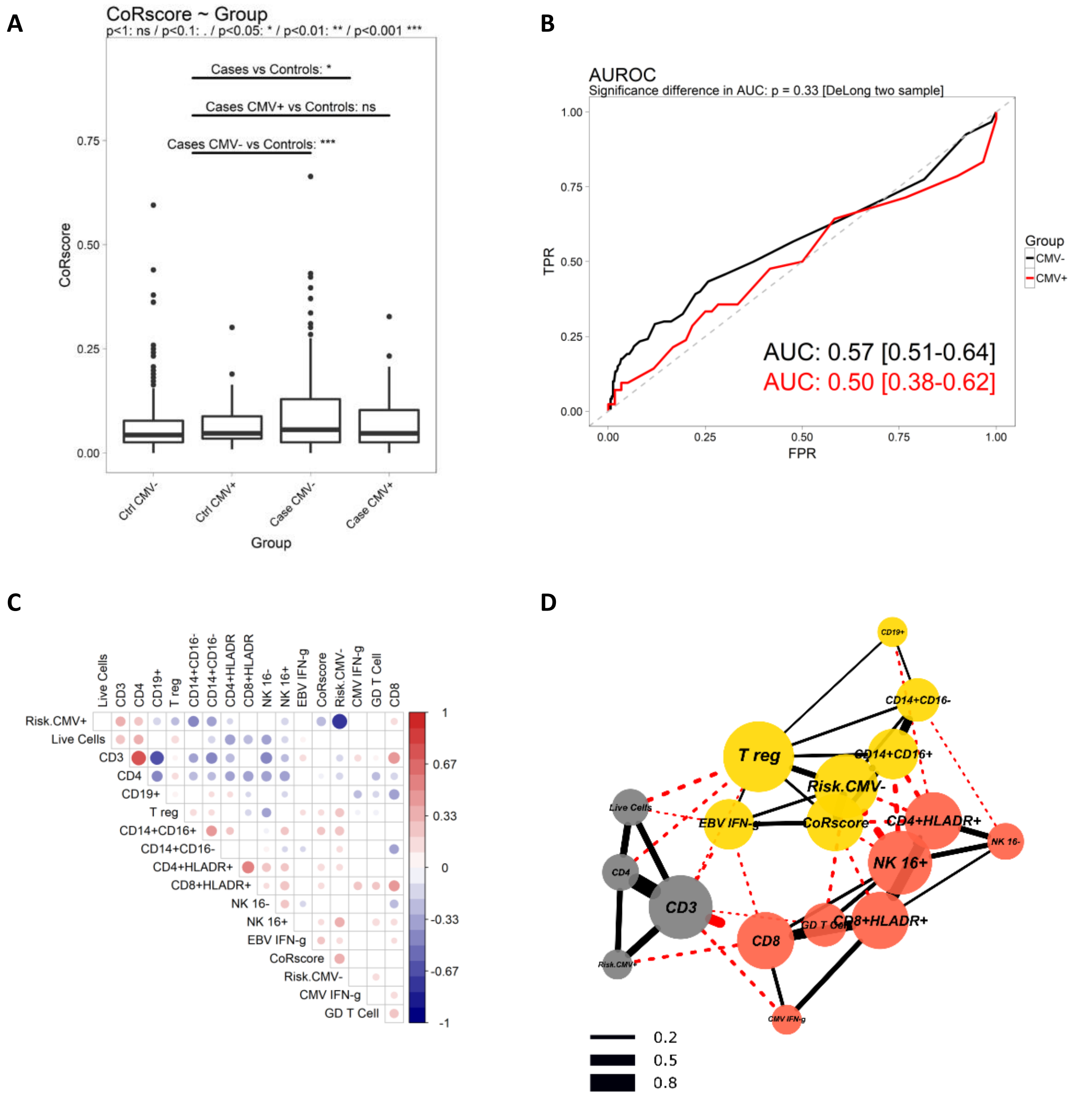

Supplementary Figure 8. **Enrichment of TB predictive Correlate of TB Risk score (CoR) from adolescents among CMV- case infants when compared to controls, but no accuracy for prediction of TB in infants**. Using a locked-down Illumina model published by Zak et al(36) for prediction of TB progression, a blinded CoR score was derived for each infant. A) After unblinding, the CoR score was found to be significantly higher in case infants when compared to controls and the greatest difference in CoR score was between CMV- case and control infants B) However, despite enrichment in case infants, the CoR score was unable to accurately classify either CMV+ or CMV- infants as cases or controls. C) The CoR score correlates with the frequency of inflammatory monocytes and the CMV- infant classifier signature. D) Network of positively correlating cell populations (spearman rho p-value <0.05) showing a cluster containing the adolescent CoR score with monocytes, EBV ELISpot and the infant CMV- classifier score. Node colour indicates community membership and red and black edges are drawn between and within communities respectively (see methods). Edge width indicates the correlation coefficient.
